# Case of Croup, in Which Tracheotomy Was Successfully Employed

**Published:** 1852-02

**Authors:** 


*A Case of Croup in which Tracheotomy was successfully employed.* By GURDON BUCK, JR. M. D.

Samuel B———, a lad eleven years of age, residing in Brooklyn, Long Island, was attacked, in the month of May, 1849, with scarlet fever, in the treatment of which calomel was freely administered. Profuse salivation succeeded, and destructive sloughing which involved the left edge of the tongue, the gums and the alveolar sockets of the lower incisor teeth of the left side, and the under lip at the left angle of the mouth. Superficial abscesses also formed beneath the scalp and upon other parts of the body. At the expiration of about five weeks from the commencement of his illness, and while gradually recovering from the debilitated condition consequent upon mercurial cachexia, he was attacked with symptoms of croup, of which he was temporarily relieved by appropriate remedies. In a few days, however, the disease re-appeared with increased violence, and, notwithstanding the judicious and skillful treatment employed, it advanced steadily toward a fatal termination. Under these circumstances, I first saw the patient on the 8th July, at 1 o'clock, P. M., at the request of his attending and consulting physicians, and found his condition as follows:

He lay in a horizontal position, with his head thrown backwards, and breathing ,with great effort, each inspiration being accompanied with a loud, hoarse, metallic sound. His countenance was anxious, his pupils dilated, and his eyes had a wild expression. The voice was reduced to a whisper. The skin was moist; the pulse, though frequent, was not irregular nor intermittent, and still retained a good degree of force. The respiratory murmur was audible and clear over the entire posterior part of the chest. The patient's situation was evidently one of imminent danger, and inasmuch as the disease had steadily advanced with only temporary abatement, during the preceding forty-eight hours, in spite of efficient treatment, the only remaining resource that afforded a reasonable hope of relief, was the operation of tracheotomy, without which it was scarcely possible for him to survive many hours. The operation was therefore decided upon without delay, and performed as follows:

A folded sheet being passed round the body, confining the arms to the sides, and the patient placed so as to expose the neck favorably to the light, a longitudinal incision, two inches and a half in length, was made over the median line, by dividing perpendicularly a transverse fold of skin, pinched up between the thumb and finger of the operator and an assistant. This incision extended over the lower half of the larynx and upper part of the trachea. The subjacent layers of the aponeurosis were then successively divided, and the sterno-hyoid and thyroid muscles drawn to either side. The isthmus of the thyroid body being now brought into view, was partly torn across at its upper edge, and partly pushed down, till the three or four superior rings of the trachea were laid bare.

After delaying till all hæmorrhage had ceased, the opening into the trachea itself was effected as follows: A transverse slit, one-fourth of an inch in length, was made between the first and second cartilaginous rings; the lower edge of the slit was then seized with a clawed forceps, and a triangular piece excised with scissors curved edgeways, the incisions commencing at either extremity of the slit, and meeting below at the inferior edge of the fourth tracheal ring. At the instant of perforating the trachea, the air rushed in with a hissing sound, and as soon as the opening was completed, respiration was promptly established through it, and in a short time became tranquil and easy. No embarrassment occurred from hæmorrhage into the trachea at the moment of opening it, the precaution having been taken to delay the opening until the flow of blood had ceased. The convulsive cough consequent upon establishing a new passage for the air to and from the lungs, was of short duration.

The rapid transition from extreme distress and anxiety to a state of tranquil repose and comfort was scarcely less gratifying to those who witnessed it, than agreeable and welcome to the patient himself. His countenance lost its wild and anxious expression, and became calm and natural. Directions were given to wipe away promptly the viscid secretion from the wound whenever coughing occurred. At 9 o'clock, P. M., we found our patient had slept quietly most of the time since the operation, and his breathing had continued perfectly easy. Fearing lest the tracheal opening should become contracted by the swelling of the edges of the wound and the accumulation of the viscid secretion around its orifice, a full sized canula of the ordinary shape was introduced and secured in place by a tape tied round the neck.

*July 9th.* Patient had passed a quiet night. Respiration continued easy, and other symptoms were favorable. Removed the tracheal tube, and, after cleansing it of the tough, viscid secretion lining the inner surface, replaced it as before.

11*th.* Progress still favorable. The rapid accumulation of the viscid secretion upon the inner surface of the tube rendered it necessary to cleanse it twice in twenty-four hours. On exploring the top of the larynx with the forefinger passed back into the fauces, the artyno-epiglottic folds were felt to be very much swollen, soft and pulpy. The epiglottis itself was normal. Applied a solution of nitrate of silver (one scruple to one drachm,) to the larynx by means of a curved whalebone probang.

12*th.* Increased the strength of the solution to one drachm to the ounce, and applied it daily. The continuance of the obstruction of the larynx without perceptible abatement showed conclusively what would have been the result of the disease if the operation had not been resorted to.

14*th.* Some diminution of the obstruction in the larynx seemed to have taken place. In closing momentarily the tracheal opening, the patient was able to breathe once or twice through the natural passage, but not without great effort.

The application of the nitrate of silver was continued till the 30th, when it was suspended for three weeks, the tube in the mean time being changed twice in twenty-four hours.

*August* 20*th.* On resuming my attendance, which had been interrupted by sickness, patient was found to have improved very much in health and appearance, and to have gained flesh and strength. The condition of the larynx was as follows:

The tube being removed and the tracheal opening closed, a few words could be uttered in an audible tone, but not without considerable effort. Respiration could be carried on through the larynx only a very short time, and also required very great effort.

With the view of exercising the obstructed parts without removing the tube, a free opening was made at the bend of the tube through its convex side, which would allow the ascending column of air to pass up through the larynx in the expiration, the outer orifice of the tube being closed.

With the tube thus arranged, and in situ, it was found that, after inflating the lungs through the tube a very considerable effort was required to expel the air through the larynx with the external orifice of the tube closed; thus showing the existence of obstruction to the egress as well as the ingress of air through the larynx. Patient was directed frequently to repeat this experiment himself, in the hope that the expansive pressure of the ascending column of air against the walls of the larynx might aid in overcoming the obstruction.

*28th.* No perceptible improvement had taken place since the preceding note. The obstruction appeared to be changed. Resumed the application of solution of nitrate of silver, (one drachm to one ounce,) and succeeded in passing the sponge into the cavity of the larynx.

*30th.* Some improvement was now observable. Patient could count from one to six in a clear tone of voice with the tube closed externally.

*September 1st.* Still further improvement has taken place. Patient could count up to thirty, and breathe a few times uninterruptedly through the larynx.

*5th.* A sudden change of weather having interrupted his improvement since the previous date, patient was now again regaining what he had lost. Ordered iodide of potassium in solution, two and a half grains, three times a day. Stopped applications to larynx.

*11th.* The improvement of the voice continued, while that of respiration did not keep pace with it; imprudent exposure to the cold wind on the roof of the house had retarded his progress. Resumed the application of solution of nitras argenti (four scruples to one ounce) to the larynx, but continued it only a few days, after which all further treatment was laid aside. Up to the present time (April 3d, 1850,) the patient, who is now submitted to your examination, has continued to enjoy excellent health. He still wears the tracheal tube arranged in the way already described, and suffers much less inconvenience from it than would be supposed. He is a boy of great activity, participates ardently in all the out-door sports of boys of his age, and also attends school. With the tube closed, he can breathe eight or ten times uninterruptedly, though before completing the number considerable effort is requisite. In using his voice he closes the tube with his finger.

In the month of October following, this patient was seen, and his condition ascertained to be very much the same as when exhibited to the Academy.—*Trans. N. Y. Academy of Medicine.*

